# High-content stimulated Raman pathology imaging and transcriptomics reveal leukemia subtype-specific lipid metabolic heterogeneity

**DOI:** 10.3389/fimmu.2025.1662281

**Published:** 2025-10-01

**Authors:** Xuelian Cheng, Jing Liu, Ming Chen, Haoyu Wang, Shuxu Dong, Yuan Zhou

**Affiliations:** ^1^ State Key Laboratory of Experimental Hematology, Institute of Hematology and Hospital of Blood Diseases, Haihe Laboratory of Cell Ecosystem, Institute of Hematology and Blood Diseases Hospital, Chinese Academy of Medical Sciences and Peking Union Medical College, Tianjin, China; ^2^ Tianjin Institutes of Health Science, Tianjin, China; ^3^ Novogene Bioinformatics Institute, Beijing, China

**Keywords:** stimulated Raman scattering, leukemia, lipid metabolism, Raman imaging, RNA sequencing

## Abstract

**Introduction:**

Leukemia, a heterogeneous group of hematological malignancies, is characterized by abnormal proliferation of immature hematopoietic cells. Current diagnostics primarily rely on morphological evaluation for subtype classification, methods that are subjective and labor-intensive. To overcome these limitations, a High-Content Spectral Raman Pathology Imaging platform (H-SRPI) was introduced.

**Methods:**

H-SRPI imaging enables profiling of proteins, nucleic acids, saturated and unsaturated lipids in leukemia. We analyzed leukemia samples from 12 patients with six distinct subtypes, alongside CD34^+^, B, T cells, monocytes and granulocytes from 3 healthy donors, by conducting high spatial resolution Raman imaging on 324 cells. We developed a single-cell phenotyping algorithm (incorporating cellular area, protein, nucleic acid, saturated and unsaturated lipid content) to distinguish leukemia subtypes. Finally, using H-SRPI and RNA-seq transcriptomics, we uncovered the critical role of lipid composition in leukemia cells across subtype classifications.

**Results:**

The single-cell phenotyping algorithm to distinguish leukemia subtypes, achieving 88.21% accuracy. H-SRPI and RNA-seq transcriptomes revealed elevated saturated and unsaturated lipid levels in acute myeloid leukemia (AML); AML-M3 favored lipid desaturation, whereas AML-M5 upregulated saturated lipid synthesis and elongation. ALL had weaker lipid metabolism characteristics than AML.

**Conclusions:**

Our study establishes H-SRPI as a label-free tool for metabolic profiling, enabling precise leukemia subclassification and revealing lipid metabolic heterogeneity as a potential therapeutic target.

## 1 Introduction

Leukemia is a clonal malignancy of hematopoietic stem and progenitor cells (HSPCs), characterized by uncontrolled proliferation, differentiation arrest, and blast accumulation (1–3). It arises from complex interactions between genetic and environmental factors. It encompasses subtypes such as: AML, ALL, chronic myeloid leukemia (CML), and chronic lymphocytic leukemia (CLL); Notably, rare variants such as prolymphocytic leukemia (PLL), large granular lymphocytic leukemia (LGL), also constitute this disease (4). AML and ALL collectively account for more than 80% of leukemia cases (5). Their heterogeneity necessitates subtype-specific management to optimize clinical outcomes. Definitive diagnosis relies on the integration of morphological, immunophenotypic, and genetic analyses (6, 7). While immunophenotypic profiling and genetic assays refine diagnostic precision, the initial morphological evaluation of bone marrow (BM) smears remains a critical starting point (3). However, morphological assessment remains inherently subjective and labor-intensive, with significant inter-observer variability—particularly when analyzing cells of identical lineage or comparable maturation stages. Therefore, it is an urgent need to develop standardized systems for precise and reproducible leukemia classification.

Raman spectroscopy is a robust label-free technique for non-destructive biomolecular characterization, enhancing analytical consistency by avoiding staining procedures (8, 9). Various studies have demonstrated the application of Raman spectroscopy in leukemia research (10–18). Some focus on elucidating metabolic changes in leukemia subtypes caused by chromosomal rearrangements and somatic mutations, while others develop novel detection platforms for subtype discrimination. Renzo et al. characterized over 300 patient-derived leukemia cells from nine subtypes using high-resolution Raman imaging (10). Our team conducted a systematic comparative analysis of leukemia subtypes versus normal cells (14). Conventional Raman systems provide both biochemical and morphological profiles for leukemia diagnostics, but they are time-consuming and limited spatial resolution. Moreover, their inherently weak scattering efficiency limits rapid imaging for clinical applications.

Stimulated Raman scattering (SRS) enables label-free chemical mapping with submicron spatial and millisecond temporal resolution by amplifying coherent anti-Stokes signals (19–22). It has been used for cancer histology, including brain, laryngeal, gastric, prostate, and breast cancer (22–26). Recent studies have demonstrated that SRS enables simultaneous detection of multiple biomolecular species, which support both precise cancer subtype classification and personalized therapy (24, 27, 28). Lipid metabolic dysregulation is proposed to underline changes in cancer cell function (29, 30). Raman imaging enables comprehensive characterization of lipid architecture, including structure, functional dynamics, and molecular composition (19, 31–33). Until now, SRS has not been applied to leukemia research.

We introduce a SRS platform in leukemia cells. By integrating SRS imaging within the C-H vibrational region (2800–3050 cm^-1^) with sparsity-constrained spectral unmixing, we mapped four major biomolecular components in leukemia cells: protein, nucleic acids, saturated lipids, and unsaturated lipids. Alterations in their absolute abundance and relative proportions were closely associated with cancer progression (22, 27, 34). In our research, high-content biochemical mapping was accomplished by a least absolute shrinkage and selection operator (LASSO) regression algorithm for spectral unmixing (22, 32). Using SRS, we examined metabolic features in leukemia blasts compared with normal counterparts. Critically, we have established a novel method for rapidly distinguishing leukemia subtypes. This suggests that cellular morphology and composition are essential for accurate diagnosis. Furthermore, we analyzed the differences in lipid metabolism between AML and ALL, as well as the lipid characteristics of different AML subtypes. Taken together, our method may enable new opportunities for accurate, rapid detection of leukemia subtypes. These findings reveal that subtype-specific dysregulation of lipid metabolism occurs and suggest potential metabolic targets for enhancing chemotherapy efficacy.

## 2 Methods and materials

### 2.1 Patients’ enrolment and standard diagnosis.

All samples were obtained from the Blood Diseases Hospital, Chinese Academy of Medical Sciences under following acquisition of informed consent from their legal guardians and/or patients authorizing the use of surplus specimens for research purposes. The study protocols were approved by the Institutional Review Board of the Institute of Hematology, Blood Diseases Hospital, PUMC/CAMS (Approval Number: NSFC2022035-EC-2). Patient characteristics are summarized in **Supplementary Table S1**. All enrolled patients underwent standardized diagnostic evaluation in accordance with the most recent WHO guidelines. To enhance morphological characterization, samples were further classified using the French-American-British (FAB) classification system. The cohort comprised 12 patients, including 4 AML subtypes (M2, M3, M4, M5) and 2 ALL subtypes: Philadelphia chromosome-negative B-cell ALL (Ph^-^) and Philadelphia chromosome-positive B-cell ALL (Ph^+^). Cellular morphology was visualized *via* MGG staining. We sorted HSPCs cells, B cells, T Cells, monocytes, and granulocytes as normal controls.

### 2.2 Leukemia BM samples preparation

The cell processing protocol was implemented according to our previously established methodology (14). Briefly, BM were processed to isolate mononuclear cells by density gradient centrifugation. Before use, cells were washed, viability assessment, and fixation in 1% paraformaldehyde (w/v).

### 2.3 Umbilical cord blood samples preparation

Samples were obtained from healthy donors (aged 20–40 years; no comorbidities) under ethical approval. Mononuclear cells were isolated by density gradient centrifugation. Following centrifugation, lymphocytes and monocytes were localized at the plasma-Ficoll-Paque interface, while granulocytes and erythrocytes were on the bottom. Monocytes were purified using magnetic bead-based separation (Miltenyi Biotec 130-050-201) according to the manufacturer instructions. We sorted HSPCs cells (CD34^+^, APC-CY7 anti-Human CD34, Biolegend 343513), B cells (CD19^+^, APC anti-Human CD19, Biolegend 302212) and T Cells (CD3^+^, FITC anti-Human CD3, BD Biosciences, 555916) by flow cytometry (BD FACSAria III). Granulocytes were isolated through hypotonic lysis using ammonium chloride solution.

### 2.4 Stimulated raman imaging

The SRS imaging experiments were performed using a multimodal nonlinear optical microscopy system (Model: UltraView MK-II, Zhendian (Suzhou) Medical Technology Co., Ltd., China). Prior to analyzing samples, reference spectra were collected from BSA, DNA, triolein, and palmitic acid samples. All images processed and analyzed using ImageJ software. The details are reported in the **Supplementary Material**.

### 2.5 Orthogonal partial least squares discriminant analysis

OPLS-DA was performed using SIMCA 14.1 software for multivariate statistical analysis. The data matrix consisted of SCR features analyzed using ImageJ software on the X-axis and cell populations on the Y-axis. Principal component analysis was employed to reduce dimensionality while maximizing variance, identifying distinct data clusters. Scores served as indispensable parameters providing biochemical insights, including key factors differentiating cell subtypes.

### 2.6 Bioinformation analysis

#### 2.6.1 Transcriptomic data acquisition and preprocessing

RNA-sequencing and microarray data of AML and ALL patients, as well as normal controls, were retrieved from public databases including TCGA, GTEx, GEO, and TARGET (see **Supplementary Methods** for dataset IDs and inclusion criteria). AML subtypes, CD34^+^ hematopoietic stem/progenitor cells (HSPCs), and healthy BM samples were selected for comparative analysis.

#### 2.6.2 Differential expression and pathway analysis

Differentially expressed genes (DEGs) were identified using the *limma* package in R. Shared DEGs across AML subtypes and between disease and control groups were intersected with predefined metabolism-related gene sets. Functional enrichment and pathway-level analysis were performed using Metascape, GSVA, and GSEA platforms. Full parameter details are provided in **Supplementary Material**.

### 2.7 Statistical analysis

Group comparisons were conducted using t-tests, ANOVA, or non-parametric alternatives based on data distribution. P-values < 0.05 were considered statistically significant. Box-and-whisker plots were used for data visualization. A detailed breakdown of statistical tests applied is available in Supplementary Material.

## 3 Results

### 3.1 Establishing SRS spectral profiles of hematopoietic cell populations

**Figure 1:**
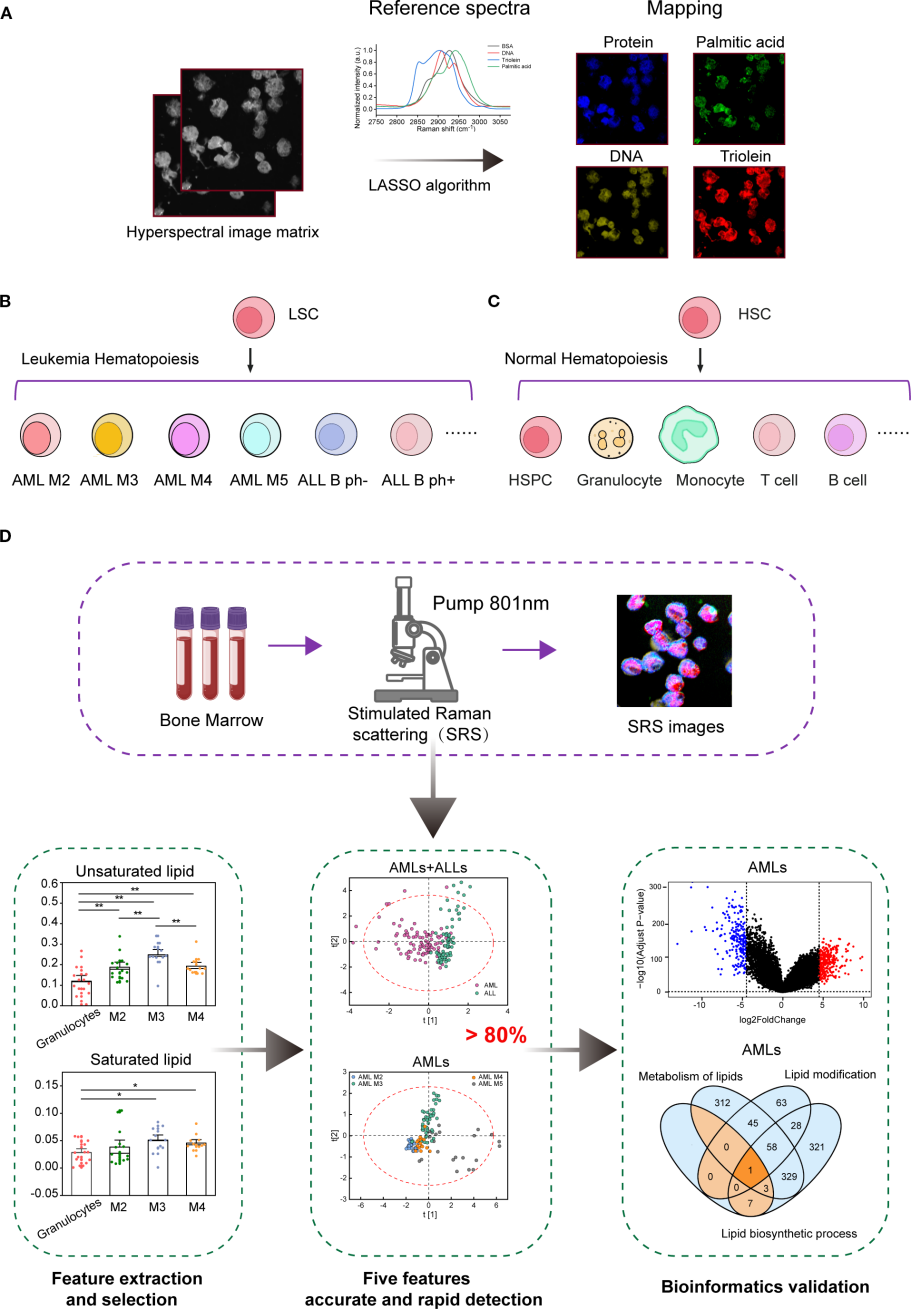
Overview of the H-SRPI principles and schematics of the experimental design. **(A)** Schematic illustration of H-SRPI mapping. Pixel-wise LASSO spectral unmixing was used to generate chemical maps based on the reference spectra of proteins, nucleic acids, saturated and unsaturated lipids. **(B)** Selected representative leukemia samples of the 6 different leukemia subtypes: AML-M2, M3, M4, M5, ALL B Ph^-^, and Ph^+^. **(C)** Selected representative normal hematopoietic samples: HSPCs, granulocytes, monocytes, T cells, and B cells. **(D)** Schematics of the experimental design.

This study evaluated the feasibility of rapid histopathological assessment of leukemic specimens using H-SRPI. As illustrated in **Figure 1A**, **Supplementary Figure 1**, the H-SRPI system generated multiplex chemical maps by targeting four key biomolecular fingerprints. Supported by multiple literature sources (22, 27, 35, 36), we used BSA, DNA, triolein, and palmitic acid samples as protein, nucleic acid, unsaturated lipid and saturated lipid standards, respectively. Reference spectra (**Figure 1A**) were derived from purified standards, and hyperspectral data were processed using LASSO regression to estimate pixel-wise biomolecular abundances. The resulting coefficients were spatially mapped to generate single-cell resolution chemical images. Acute leukemia was selected as the disease model. BM were collected with six distinct subtypes: AML-M2, M3, M4, M5 and B-ALL Ph^-^, Ph^+^ (**Figure 1B**), all of which are believed to originate from leukemic stem cells (LSCs). The healthy controls were containing: HSPCs, granulocytes, monocytes, B cells, and T cells (**Figure 1C**). As shown in **Figure 1D**, the workflow of H-SRPI is described.

### 3.2 H-SRPI imaging enables profiling of proteins, nucleic acids, and lipids in AML

**Figure 2:**
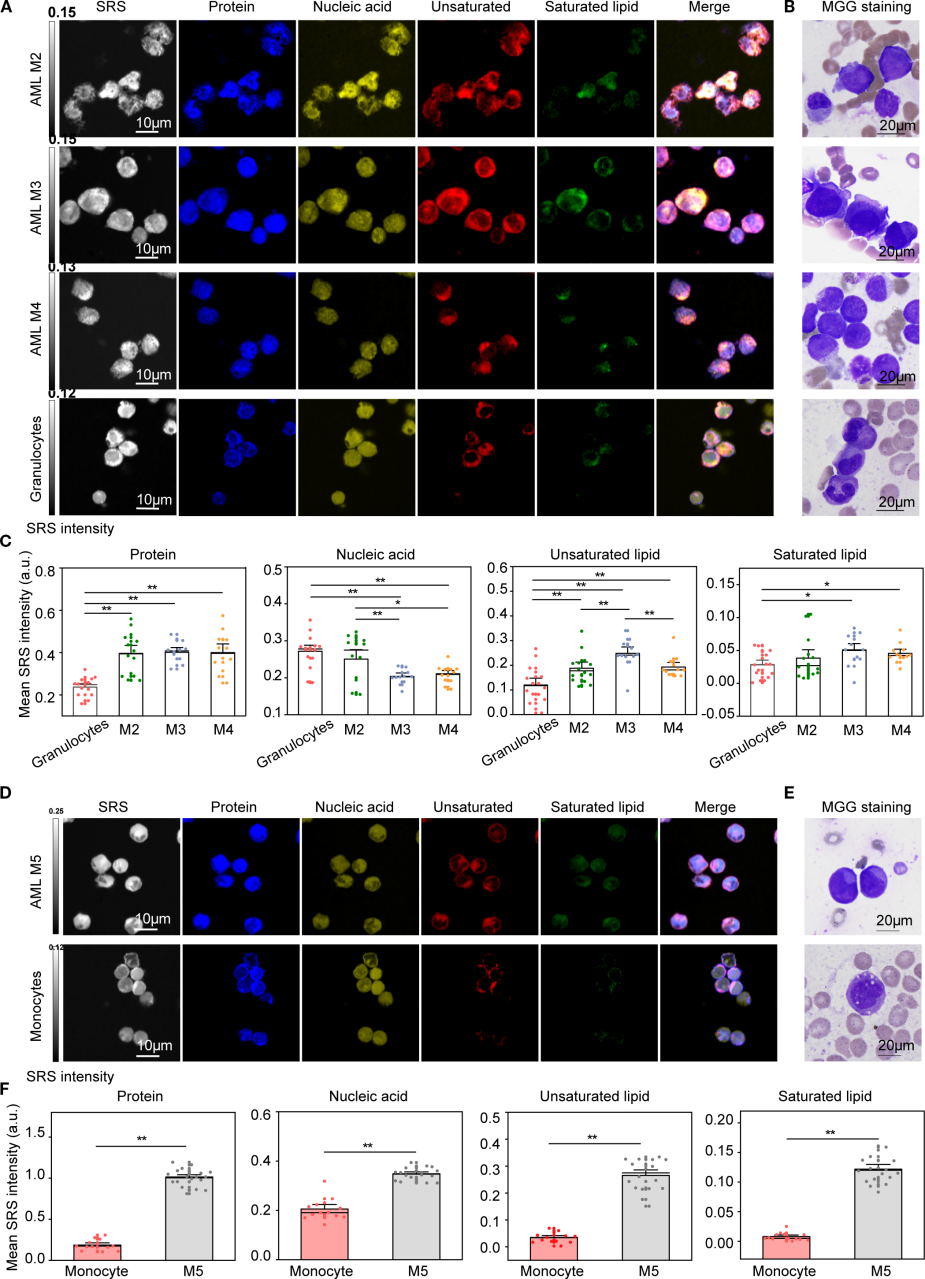
H-SRPI and MGG imaging reveal distinct intracellular carbohydrates in AML cells. **(A)** Representative H-SRPI images showing the distribution of proteins (blue), nucleic acids (yellow), unsaturated lipids (red), and saturated lipids (green) in AML-M2, M3, M4 cells and normal granulocytes. **(B)** MGG staining of corresponding cells highlights morphological differences between leukemic blasts and granulocytes. **(C)** Quantitative analysis of H-SRPI mapped signal of proteins, nucleic acids, unsaturated lipids, and saturated lipids between leukemic blasts and granulocytes. **(D, E)** Representative H-SRPI and MGG images of AML M5 cells and monocytes. **(F)** Quantitative analysis of H-SRPI mapped signal from **(D)**. *P < 0.05, **P < 0.01; a.u., arbitrary units.

Metabolic reprogramming and epigenetic remodeling are hallmarks of leukemogenesis and AML disease progression (37, 38). These alterations are often genotype-specific and accompanied by epigenetic and functional changes that promote oncogenic pathway activation (39, 40). To evaluate the utility of H-SRPI in capturing these metabolic shifts, cross-comparative analysis between leukemia blasts and their normal counterparts was to conduct delineate malignancy-associated biochemical signatures. The purity of the three populations exceeded 95% by flow cytometry, as reported in **Supplementary Figure 2**. We first compared granulocyte-matched cells. Given the granulocytic dominance in AML M2, M3, and M4, these subtypes were analyzed against normal granulocytes. H-SRPI imaging (**Figure 2A**) revealed leukemic cells exhibited significantly elevated protein and lipid signals, suggesting enhanced biosynthetic activity, MGG staining (**Figure 2B**) confirmed the presence of abnormal myeloid blasts. And quantitative analysis (**Figure 2C**) showed significantly higher levels of proteins and lipids in AML-M2, M3, M4 cells. Notably, the increase in unsaturated lipids exceeded that of saturated lipids, indicating a preferential shift toward unsaturated lipid biosynthesis. In contrast, nucleic acid content was significantly decreased, consistent with reduced DNA abundance during leukemogenesis.

We next compared M5 cells with monocytes, given their shared monocytic lineage. H-SRPI imaging (**Figure 2D**) revealed consistently elevated levels of proteins, nucleic acids, and lipids in M5 cells. These differences were further supported by MGG staining of both monoblasts and monocytes (**Figure 2E**). Quantitative analysis (**Figure 2F**) demonstrated a substantial increase in all four biomolecular components in AML M5 cells, reflecting robust metabolic reprogramming characterized by enhanced protein synthesis and lipid accumulation.

### 3.3 H-SRPI imaging discloses metabolic features in B-ALL cells

**Figure 3:**
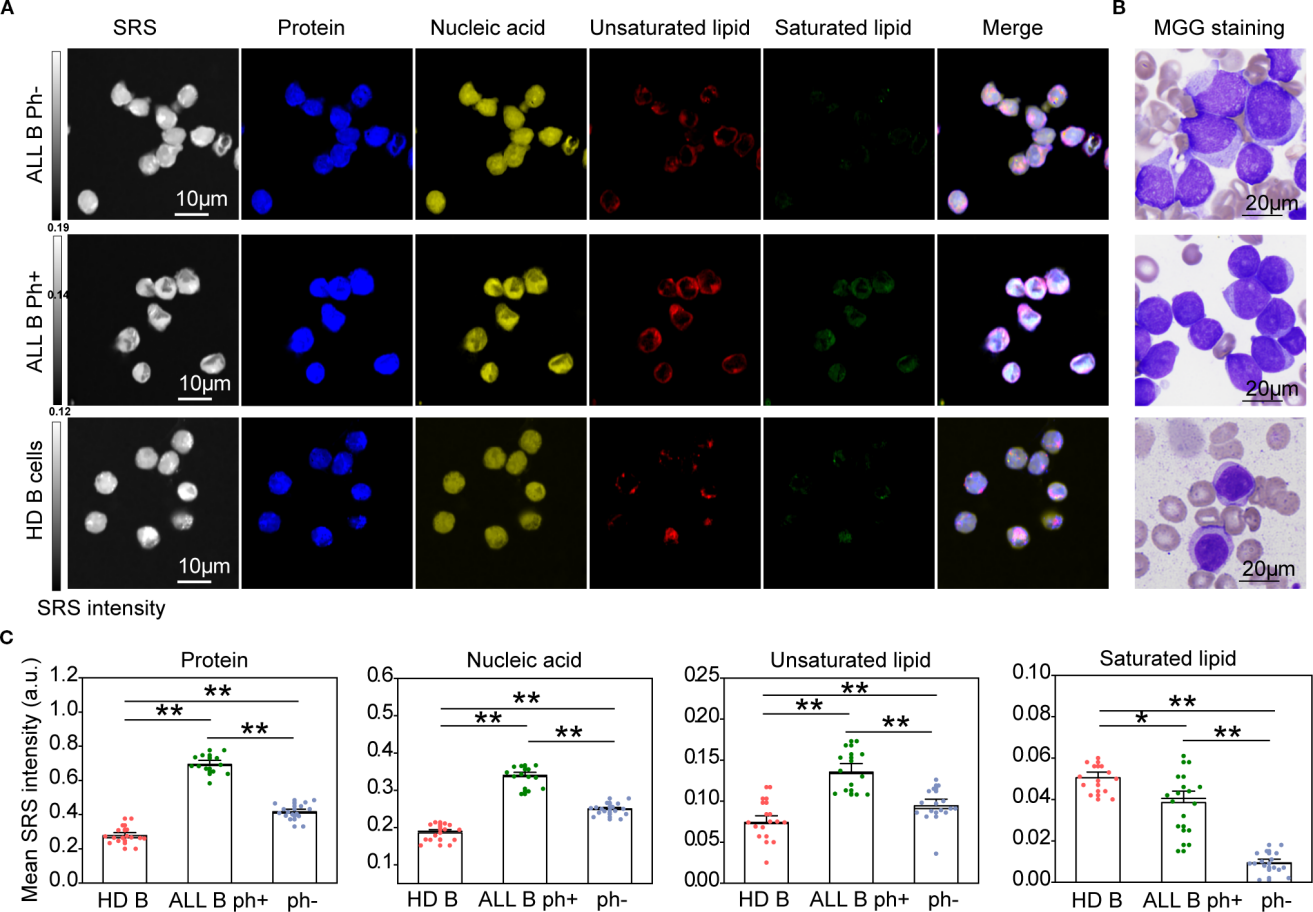
H-SRPI and MGG imaging reveal biomolecular composition in ALL cells. **(A)** Representative H-SRPI and multichannel images showing distributions of proteins (blue), nucleic acids (yellow), unsaturated lipids (red), and saturated lipids (green) in Ph^+^ and Ph^-^ B-ALL, and HD B cells. Merged images demonstrate intracellular localization patterns. **(B)** MGG staining reveals morphological differences among the three groups. **(C)** Quantitative analysis of H-SRPI signal intensities for proteins, nucleic acids, unsaturated lipids, and saturated lipids. *P < 0.05, **P < 0.01; a.u., arbitrary units.

To systematically compare biomolecular profiles between ALL and normal B cells, we compared ALL B cells with healthy donors (HD) B cells. H-SRPI revealed distinct intracellular distributions: both Ph^+^ and Ph^-^ B-ALL cells exhibited elevated proteins, nucleic acids, and unsaturated lipids compared to HD B cells, with the Ph^+^ group showing the highest enrichment of unsaturated lipids (**Figures 3A, C**). These molecular distinctions were corroborated by MGG staining, which highlighted clear morphological distinctions across groups (**Figure 3B**). However, in comparison to AML cells, ALL cells demonstrated weaker Raman signals and markedly reduced lipid content, suggesting that protein and nucleic acid metabolism dominate in ALL cell physiology.

### 3.4 Identification of leukemia subtypes by OPLS-DA algorithm

**Figure 4:**
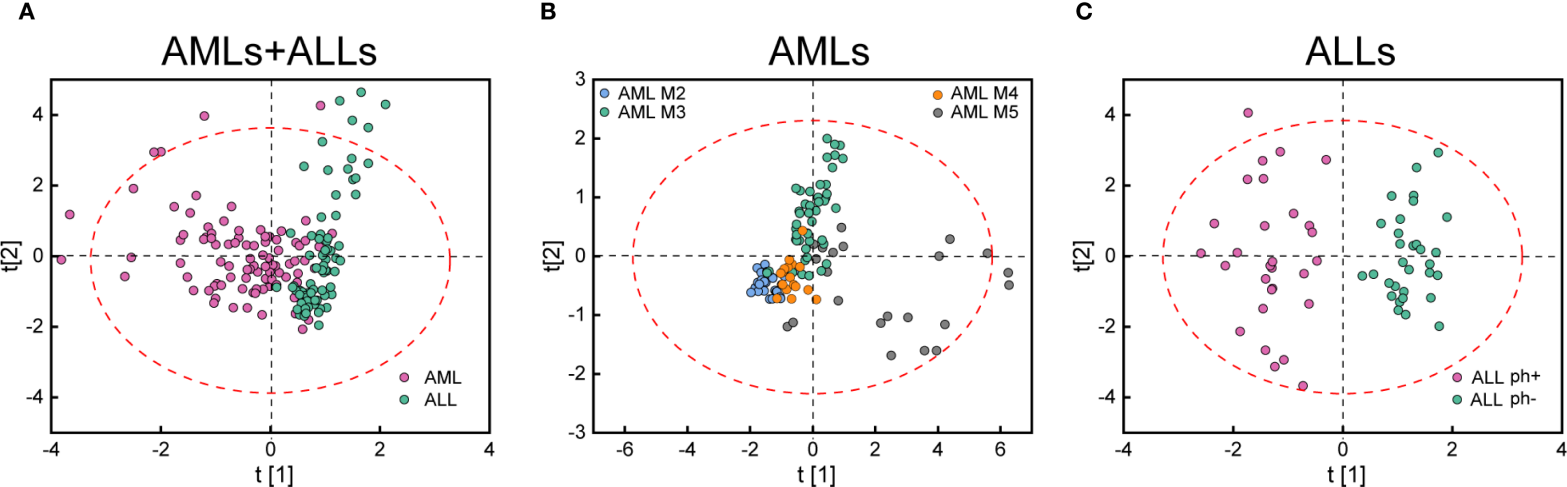
Measurement and dimension reduction of five SRS features (cellular area, protein, nucleic acid, saturated and unsaturated lipid content) of hematopoietic cells. The scores plot of OPLS model for **(A)** AMLs+ALLs, **(B)** AMLs, and **(C)** ALLs.

We quantitatively characterized 174 individual cells obtained from 12 leukemia patients. 5 features of cellular area and chemical composition (protein, nucleic acid, saturated and unsaturated lipid content) were extracted by ImageJ software. Nevertheless, the differences of composition and morphology features between leukemia subtypes were not very evident, which was likely due to the existence of heterogeneous populations in each specimen. Therefore, we proposed to analyze the features by OPLS-DA. The resulting scatter plots, reporting the scores of the first two canonical variables, are shown in **Figures 4A–C**. As shown in **Figures 4A** and **Supplementary Figure 3A**, representing scores of “AMLs+ALLs”, a certain degree of separation was obtained between AML and ALL subtypes (AML: 90% precision and 90% sensitivity; ALL: 81% precision and 80% sensitivity).

When only AML subtypes are considered, OPLS-DA can separate AML M2 (100% precision and 77% sensitivity), M3 (85% precision and 89% sensitivity), M4 (92% precision and 63% sensitivity), and M5 (72% precision and 96% sensitivity) cells (**Figures 4B**; **Supplementary Figure 3B**). The confusion matrix resulting shows very good accuracy for the classification of M5 and other subtypes. M2, M3, and M4 cluster together due to their similarity as granulocytes. When only ALL subtypes are analyzed, a very good separation can be seen between Ph^−^ and Ph^+^ (100% precision and 100% sensitivity) (**Figures 4C**; **Supplementary Figure 3C**). The total accuracy is 88.21%. These results suggest that our H-SRPI imaging method is capable to detect leukemia cells with high sensitivity and specificity.

### 3.5 Differences in lipid metabolism characteristics between AML and ALL

**Figure 5:**
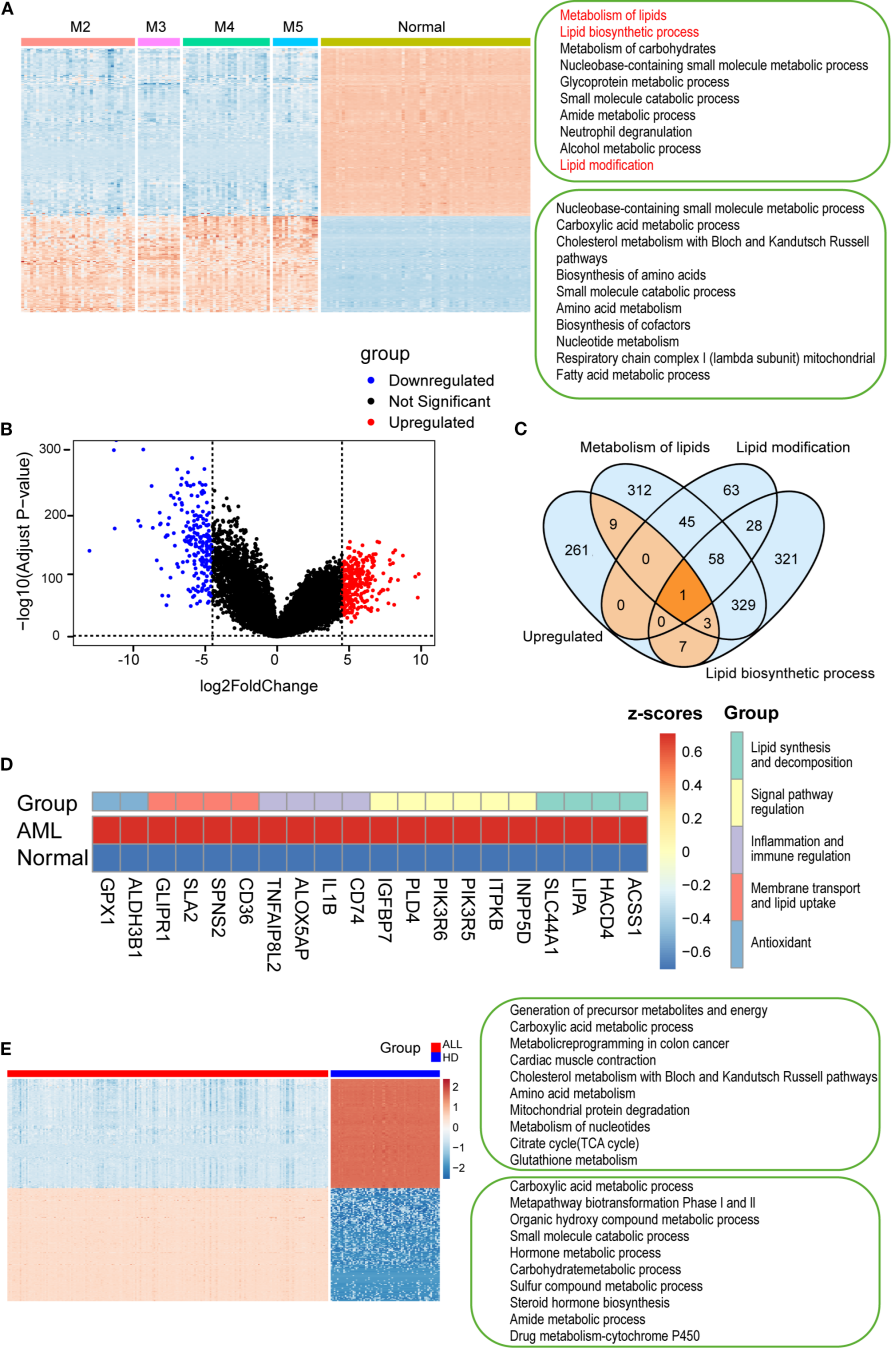
Transcriptomic analysis of AML and ALL cells from the TCGA datasets. **(A)** Heatmap showing gene expression in AML subtypes and normal cells. Lipid-related pathways specifically activated in AML are highlighted in red). **(B)** A volcano plot of differentially expressed genes between AML and normal samples. **(C)** Venn diagram identifying 20 upregulated genes involved in lipid metabolism, biosynthesis, and modification. **(D)** Heatmap of the 20 genes, categorized by function: lipid metabolism, transport, signaling, immunity, and antioxidant activity (color-coded). **(E)** Heatmap showing differentially expressed genes between B-ALL and HD B cells, with enrichment analysis of top 10 pathways in each group (Metascape). P < 0.01, P < 0.001, by one-way ANOVA with *post hoc* test.

To investigate lipid compositional alterations in AML, we analyzed the transcriptomes of AML samples. Gene enrichment analysis using Metascape and GSEA revealed significant upregulation of lipid-associated pathways in AML, including lipid metabolism, biosynthesis, and modification, along with carbohydrate and small molecule metabolic processes (**Figure 5A**; **Supplementary Figures 4A–D**). By contrast, genes enriched in normal samples were mainly associated with protein and nucleic acid-related pathways. Notably, none of the top ten pathways in normal cells were lipid-related.

To further characterize these metabolic changes, we identified 281 significantly upregulated genes in AML cells (**Figure 5B**). Venn diagram analysis revealed overlapping genes involved in lipid metabolism, lipid biosynthesis, and lipid modification (**Figure 5C**), suggesting their involvement in AML-specific lipid reprogramming. 20 upregulated genes were identified within the intersection of lipid metabolism-related pathways in AML cells (**Figure 5D**). Among them, ACSS1, HACD4, LIPA, SLC44A1, and CD36 were particularly notable due to their central roles in lipid synthesis, elongation, degradation, and transport. ACSS1 and HACD4 mediate acetate utilization and very long-chain fatty acid elongation, respectively, reflecting enhanced anabolic lipid metabolism in AML (41, 42). CD36, a key fatty acid transporter (43), was significantly upregulated and correlated with the elevated unsaturated lipid levels observed by H-SRPI imaging. These findings underscore the role of lipid metabolic reprogramming in AML, as the 20 upregulated genes enhanced metabolic flexibility and leukemic progression, and may facilitate drug resistance and immune evasion.

Next, we analyzed the metabolic characteristics of ALL cells. It showed enrichment in pathways such as the carboxylic acid metabolic process, metapathway biotransformation Phase I and II, whereas normal B cells were enriched in energy-generating and biosynthetic pathways (**Figure 5E**). Notably, lipid metabolism–related pathways were absent from the top enriched terms in both groups. Consistently, GSEA results indicated lipid-associated pathways remained non-enriched (**Supplementary Figure 4E**), while significant upregulation of cyclic nucleotide metabolic process and CGMP metabolic process (**Supplementary Figure 4F**). These findings verified that ALL cells rely more heavily on protein and nucleic acid metabolism than on lipid metabolism.

### 3.6 H-SRPI discloses metabolic profile reprogramming of lipid unsaturation in AML

**Figure 6:**
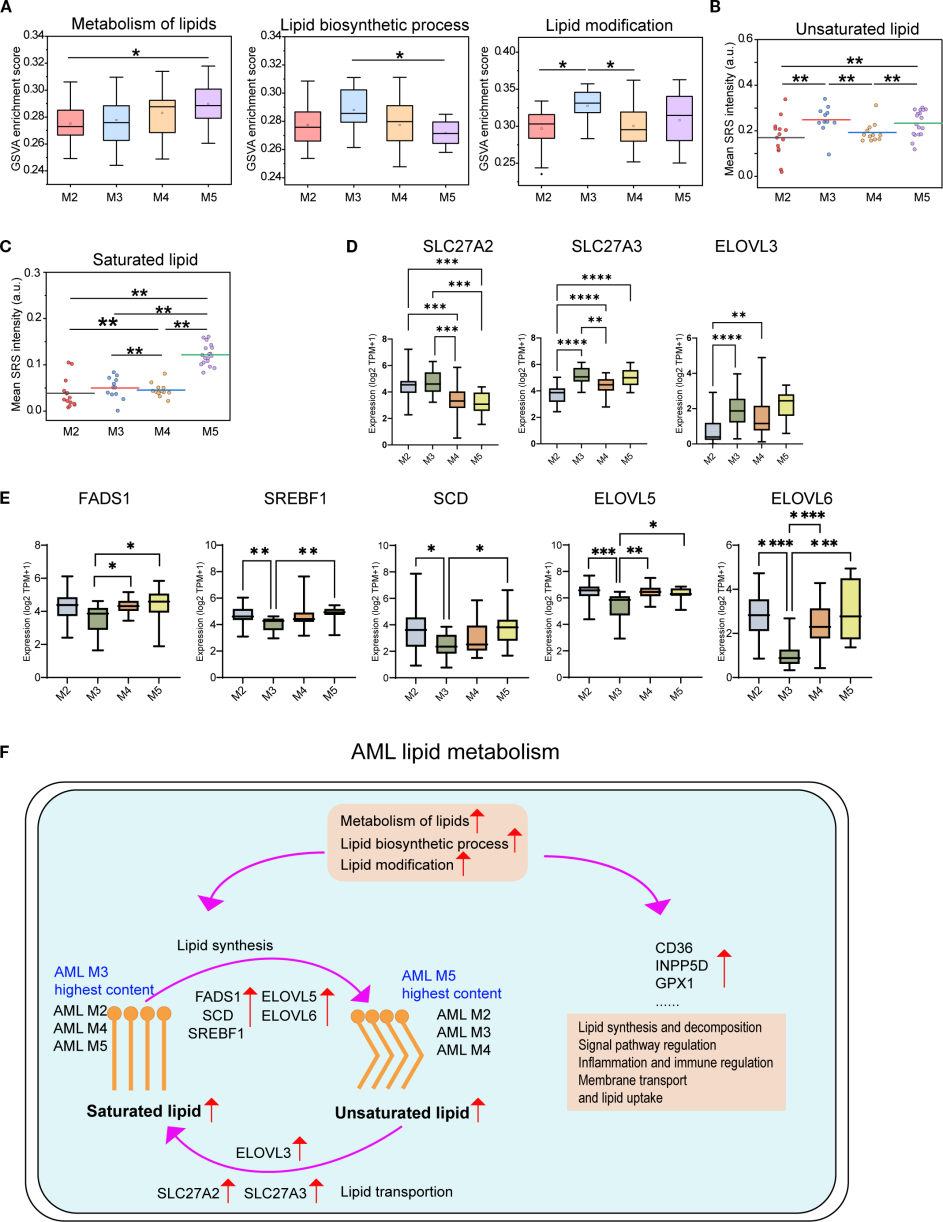
Comparison of lipid metabolism pathways and gene expression across the four AML subtypes. **(A)** GSVA scores for lipid-associated pathways across AML subtypes (M2–M5). Quantification of unsaturated **(B)** and saturated **(C)** lipid levels in AML cells by H-SRPI imaging. **(D)** Expression levels of fatty acid transport and elongation genes (SLC27A2, SLC27A3, and ELOVL3). **(E)** Expression levels of genes involved in fatty acid synthesis, desaturation, and elongation (FADS1, SREBF1, SCD, ELOVL5, and ELOVL6). Statistical comparisons were performed using one-way ANOVA with *post hoc* tests. *P < 0.05; **P < 0.01; ***P < 0.001; ****P < 0.0001. **(F)** Schematic of the lipid metabolism model of AML. Schematic representation of altered lipid metabolism in AML, highlighting subtype-specific differences in saturated and unsaturated lipid content. Key regulatory genes involved in lipid synthesis (FADS1, SCD, ELOVL5/6, SREBF1), transport (SLC27A2/3, ELOVL3), and uptake (CD36, INPP5D, GPX1) are indicated. Enriched pathways include lipid biosynthesis, modification, and immune regulation.

Accumulating evidence indicates that AML cells undergo extensive lipid metabolic reprogramming to sustain malignant proliferation and survival (44). To investigate subtype-specific lipid metabolic adaptations, we performed GSVA across four AML subtypes. M5 showed the highest scores in overall lipid metabolism, while M3 displayed the strongest enrichment in lipid biosynthetic and modification pathways (**Figures 6A-C**), indicating distinct metabolic strategies. To elucidate underlying mechanisms, we analyzed the expression of genes involved in fatty acid transport, activation, synthesis, desaturation, and elongation. M3 cells showed high expression of SLC27A2,SLC27A3, fatty acid transport protein (FATP) family that mediate FA uptake for β-oxidation (45), along with elevated ELOVL3 (46) (**Figure 6D**). In contrast, M5 cells upregulated lipogenic genes (**Figure 6E**), including FADS1, SREBF1 (lipogenesis regulator driving ACLY, FASN, ACACA, and SCD (47)). SCD facilitates FA desaturation to support membrane dynamics (48). ELOVL5/6 extend PUFA and SFA chains to modulate lipid composition (46). Taking together, these results demonstrate distinct lipid metabolic programs in AML. The M3 favors lipid desaturation and unsaturation, whereas the M5 upregulates saturated lipid synthesis and elongation, forming a metabolic axis linked to leukemic progression and therapeutic response. To investigate lipid metabolism during AML progression process, we analyzed HSPCs and aberrant promyelocytes from M3-AML. M3 exhibited significantly higher levels of unsaturated lipids content and metabolism features (**Supplementary Figure 5**).

## 4 Discussion

Through multimodal integration of H-SRPI, MGG staining, and RNA-seq transcriptomics across six leukemia subtypes and five normal hematopoietic cell types, we uncovered the critical role of lipid composition remodeling in leukemia subtype differentiation. As the first application of H-SRPI in leukemia research, our approach enabled high-resolution imaging with clear morphological distinction, establishing a novel framework for precise subtype identification. Multimodal imaging of both saturated and unsaturated lipids improved diagnostic sensitivity and revealed distinct lipid metabolic patterns across subtypes. Simultaneously, we systematically profiled AML lipid metabolism (**Figure 6F**), highlighting lipid metabolic reprogramming as a hallmark of leukemia progression and a promising target for translational therapy.

First, our study established a stain-free H-SRPI platform for *in situ* pathological diagnosis of leukemia cells at single-cell resolution. Compared to the conventional MGG staining, H-SRPI provided rich chemical information, particularly enabling clear distinction between saturated and unsaturated lipids profiles. H-SRPI holds promise for enhancing leukemia classification accuracy, achieving 84.21% accuracy, which is essential for guiding clinical decision-making.

Second, our study highlights the metabolic reprogramming of lipid concentrations in leukemia cells. Altered lipid metabolism has been implicated in leukemia cell function (49), our results showed that AML cells exhibit elevated levels of both saturated and unsaturated lipids to support increased demands for membrane synthesis and remodeling, disrupting lipid and membrane homeostasis. In contrast to AML cells, ALL cells exhibited markedly altered lipid composition, suggesting a distinct metabolic profile. This discrepancy may be driven by higher fatty acid synthase *(FASN)* expression and activation of lipid synthesis pathways (e.g., PI3K/AKT/mTOR) in AML (50), which are comparatively less active or absent in ALL, resulting in reduced lipid biosynthesis. Our study highlights the therapeutic potential of regulating lipid homeostasis for leukemia treatment. Investigating lipid metabolic heterogeneity across leukemia subtypes may inform the development of lipid-targeted therapies with translational relevance.

Third, our platform can accurately quantify MPO, an oxidative enzyme constituting 3-5% of total protein in mature granulocytes (51). Given the prognostic relevance of antioxidant enzymes in leukemia, we compared MPO levels across granulocytes, monocytes, B cells, and T cells (**Supplementary Figure 6**). As expected, MPO is highly expressed in granulocytes, with minimal expression observed in other cell types, confirming the high sensitivity of H-SRPI for detecting low-abundance, labile enzymes and expanding its potential applications. Furthermore, MPO may serve as an additional marker for leukemia classification to improve the accuracy of pathological detection.

In this study, we identified four major components to capture the major Raman signals within a cell. Since BSA exhibits methyl and methylene group vibrational frequencies similar to those found in vertebrate proteomes (20), it was used as a protein reference. Purified DNA from normal BM cells served as the nucleic acid reference. Triolein and palmitic acid, which are abundant and biologically significant cellular lipids, were employed as lipid references, consistent with their common use in SRS imaging studies (22, 27). We acknowledge that these standard molecules may not perfectly reflect the diversity of molecular subtypes present in the sample. Therefore, more precise spectral isolation techniques will be employed for further refinement. On the other hand, we note that the 2800–3050 cm^-1^ region exhibits substantial peak overlap between proteins and nucleic acids, which can blur nuclear–cytoplasmic boundaries in SRS images. Although Raman fingerprint bands are information-rich, their small cross sections result in noisy measurements, making it difficult to distinguish less abundant metabolites from background noise. The high wavenumber C–H bands (2800–3050 cm^-1^) can mitigate this sensitivity issue due to their significantly larger cross sections compared to fingerprint bands. However, all major metabolic species—proteins, nucleic acids, and lipids—exhibit essential yet overlapping Raman peaks in this region. Existing hyperspectral data analysis methods cannot fully capture the rich information content of C–H vibrations due to significant cross-talk among the resulting chemical maps (22, 27).

Collectively, our work demonstrates the potential of H-SRPI to reveal biological heterogeneity in leukemia cells, elucidate the role of altered lipid composition in leukemogenesis, and provide a novel approach to leukemia diagnosis and treatment. In summary, we propose that Raman spectroscopy has evolved into an increasingly powerful toolkit for biologists and clinicians, delivering molecule-specific insights at the single-cell level with expanding capabilities at the subcellular scale.

## Data Availability

The original contributions presented in the study are included in the article/**Supplementary Material**. Further inquiries can be directed to the corresponding author/s.
