# Supplementary material for: High-content stimulated Raman pathology imaging and transcriptomics reveal leukemia subtype-specific lipid metabolic heterogeneity
Source: Front Immunol. 2025 Oct 1;16:1662281. doi: 10.3389/fimmu.2025.1662281 (PMC12520939; doi:10.3389/fimmu.2025.1662281)
Supplement: Supplementary file 1 [file DataSheet1.docx]

Supplementary Material

# Supplementary Methods

# Stimulated Raman imaging

The SRS imaging experiments were performed using a multimodal nonlinear optical microscopy system (Model: UltraView MK-II, Zhendian (Suzhou) Medical Technology Co., Ltd., China) (**Supplementary Figure 1**). The UltraView microscope was equipped with an InSight×3 femtosecond laser (Spectra-Physics/Newport, pulse width < 120 fs), a spectral focusing module, and a laser scanning microscope to acquire hyperspectral SRS images. During experiments, the wavelengths of the pump and Stokes beams were tuned to 801 nm and 1045 nm, respectively, covering the spectral window of 2750-3030 cm^-1^. Prior to analyzing BM samples, reference spectra were collected from BSA, DNA, triolein, and palmitic acid samples. These reference spectra were subsequently used for spectral unmixing of BM cell images. Both reference samples and BM samples were sandwiched between two coverslips, fixed with UV-curable adhesive, and mounted on the microscope for imaging.  A 60× oil-immersion objective lens (UPlanXAPO60XO, NA=1.42; Olympus, Japan) was employed for image acquisition, with each pixel scanned for 10 μs and averaged over five iterations to enhance signal fidelity. All images were subsequently processed and analyzed using ImageJ software, while spectral unmixing was conducted with SpecFinder software provided by Zhendian (Suzhou) Medical Technology. During SRS imaging, laser power was maintained at 20 mW for the pump beam and 100 mW for the Stokes beam at the entrance port of the laser scanning microscope.

# Bioinformation analysis

# Data acquisition and processing

RNA-sequencing data and associated clinical information of patients with AML were retrieved from The Cancer Genome Atlas (TCGA) database. A total of 151 AML samples were initially included. For this study, we selected cases with “leukemia French American British morphology code” classified as M2, M3, M4, and M5, based on the FAB classification system, to ensure morphological consistency of the included AML subtypes.

As a normal control group, RNA-sequencing data of healthy BM samples (n=90) were acquired through the Genotype-Tissue Expression (GTEx) database. The combined TCGA and GTEx data were used for subsequent analyses, including differential gene expression and classification modeling. Gene expression profiles of the GSE9476 dataset, profiled using the Affymetrix U133A Array platform, along with clinical annotations were extracted from the GEO (Gene Expression Omnibus) repository. For this study, only samples annotated as CD34-positive (CD34^+^) in the clinical data were included for further analysis. Transcriptomic and phenotype data for ALL cases were accessed via the Therapeutically Applicable Research to Generate Effective Treatments (TARGET) database via the UCSC Xena browser. A total of 206 samples with a negative BCR::ABL fusion gene status, as indicated in the phenotype data, were selected for further analysis.

# Differential expression analysis

The ‘limma’ package (version 3.62.1) in R (version 4.4.2) was utilized to perform differential expression analysis. Each AML subtype (M2, M3, M4, and M5) was individually compared with normal bone marrow samples from the GTEx database. A threshold of |log2 fold change| > 2 combined with an adjusted p-value < 0.05 was used to identify genes with statistically significant differential expression. A common set of dysregulated genes was obtained by intersecting the differentially expressed gene sets identified in each of the four AML subtypes. In addition to the subtype comparisons, differential expression analysis was also conducted between ALL samples and HD, using a more stringent threshold of |log_2_ FC| > 7 combined with an adjusted p-value < 0.05. Moreover, gene expression differences between the M3 subtype and HSPCs were analyzed using a threshold of |log_2_ FC| > 1 and adjusted p-value < 0.05. Volcano plots were generated using the ggscatter function from the “ggpubr” R package to visualize DEGs from the global AML versus normal comparison (cutoff: |log_2_ FC| ≥ 4.5, adjusted p-value < 0.05) and the M3 versus CD34^+^ comparison (cutoff: |log_2_ FC| ≥ 3, adjusted p-value < 0.05).

# Identification of metabolism-related DEGs

Shared DEGs across AML subtypes were overlapped with a curated list of metabolism-related genes (1) using the Venny tool, yielding 258 metabolism-associated DEGs. Heatmaps of gene expression were generated using the “pheatmap” R package. Additional intersections were performed between metabolism-related genes and DEGs identified from ALL vs. normal, and M3 vs. HSPCs comparisons, with corresponding heatmaps plotted.

# Functional enrichment analysis

To investigate the biological implications of metabolism-related DEGs, intersected gene sets were categorized based on the direction of regulation and subjected to enrichment analysis. Specifically, the 258 metabolism-related DEGs obtained from the intersection of M2, M3, M4, and M5 AML subtype DEGs with predefined metabolic gene sets were stratified into upregulated (log_2_ FC> 2) and downregulated (log_2_ FC< -2) groups. Similarly, 301 metabolism-related DEGs derived from the comparison between ALL samples and HD were grouped based on log_2_ fold change direction. In addition, the intersection of DEGs from the M3 subtype versus HSPCs cells and metabolic gene sets resulted in 1008 metabolism-associated DEGs, which were likewise categorized into upregulated and downregulated subsets. Each of these gene groups was independently analyzed for functional enrichment using the Metascape platform. The analysis was performed with the following settings: species set to *Homo sapiens*, analysis type set to “Express Analysis”, and all other parameters kept at default.

# Gene set variation analysis (GSVA)

The “GSVA” R package was applied to estimate enrichment scores for pathways based on the expression levels of upregulated metabolism-related genes. Using GraphPad Prism 9.5 (GraphPad Software, CA), box-and-whisker plots were created to depict enrichment score distributions for AML versus normal cohorts. Boxes represent the 25th to 75th percentile range, the median is shown as a central line, and whiskers indicate the minimum and maximum data points. This visualization effectively illustrates the distribution and variability of pathway activity between the two groups. To identify genes associated with both metabolic pathways and AML progression, the GSVA-enriched gene sets were intersected with the 287 upregulated genes identified in AML. The overlap of these gene sets was visualized using a Venn diagram, and the expression patterns of the 20 overlapping genes were illustrated using a heatmap generated with the “pheatmap” package in R. Similarly, GSVA was conducted to the comparison between M3 subtype and CD34^+^ cells. The enriched gene sets were intersected with 109 upregulated genes from this comparison, and the expression profiles of the overlapping genes were also visualized in heatmaps using the “pheatmap” package, following same approach as described above.

# Gene set enrichment analysis (GSEA)

Gene set enrichment analysis was conducted using the standalone software (version 4.3.2) provided by the Broad Institute. The analysis was performed using a gene expression matrix paired with a corresponding phenotype label file, which defined the experimental groups (e.g., ALL versus normal). Gene sets were selected from the Gene Ontology Biological Process (GO BP) collection in the MSigDB database, specifically including “GO BP CYCLIC NUCLEOTIDE METABOLIC PROCESS”, “GO BP CGMP METABOLIC PROCESS”, and “GO BP REGULATION OF LIPID CATABOLIC PROCESS”.

A total of 1,000 permutations were applied, with “phenotype” labels used as the permutation basis. Gene sets were considered significantly enriched if they satisfied any of the following thresholds: nominal p-value < 0.05, family-wise error rate (FWER) p-value < 0.05, or false discovery rate (FDR) q-value < 0.25. The normalized enrichment score (NES), nominal p-value or FWER p-value, and FDR q-value were used to evaluate the significance and magnitude of enrichment. Enrichment plots and summary bar charts were exported from the GSEA desktop application for downstream visualization and interpretation.

# Statistical analysis

GraphPad Prism v9.5 (GraphPad Software, CA, USA) was used to conduct all statistical analyses. Normal and log-normal distribution assumptions were tested to evaluate data distribution. When comparing two independent groups with normal distribution, a two-tailed unpaired Student’s t-test was applied. Homogeneity of variances was evaluated using the F-test; if equal variances were assumed, the standard Student’s t-test was applied, whereas Welch’s correction was used when variances were unequal. For non-normally distributed data, the Mann–Whitney U test was performed.

For comparisons involving more than two groups, such as among AML subtypes (M2, M3, M4, and M5), data were first assessed for normality and homogeneity of variances. When both criteria were satisfied, one-way ANOVA followed by Tukey’s multiple comparisons test was performed. In cases where data were normally distributed but exhibited unequal variances, Brown-Forsythe and Welch’s ANOVA tests were applied. The Kruskal–Wallis test was used for non-normally distributed data. Visualization of results was conducted using box-and-whisker plots, where the boxes represent the interquartile range (25th–75th percentile), the horizontal line denotes the median, and whiskers indicate the minimum and maximum values. Statistical significance was determined at *p* < 0.05.

# Supplementary Figures and Tables

## Supplementary Figures


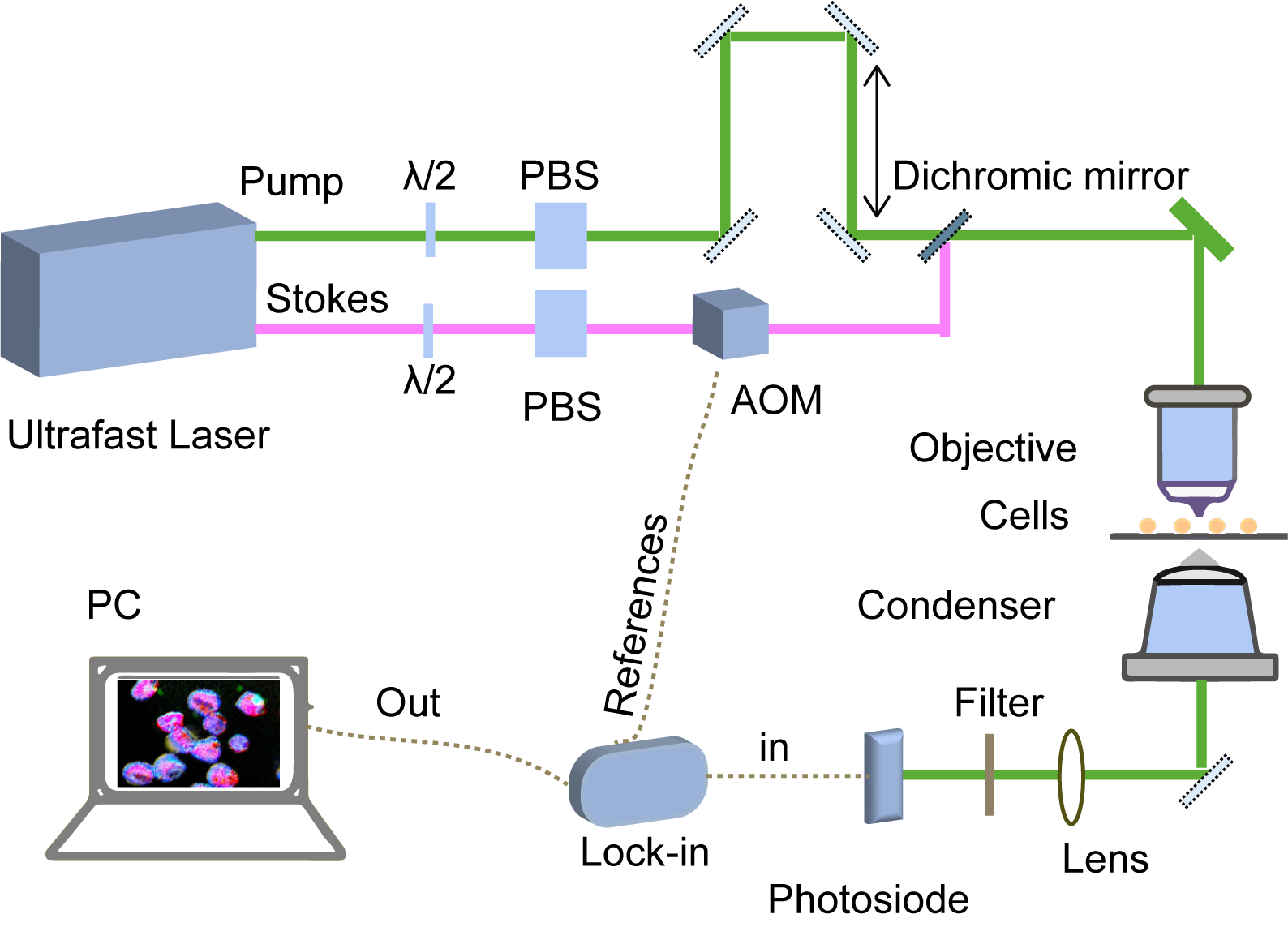


**Supplementary Figure 1**. Schematic of the high-content spectral raman microscope (H-SRPI). The system includes a state-of-the-art SRS imaging system equipped with an InSight×3 femtosecond laser.


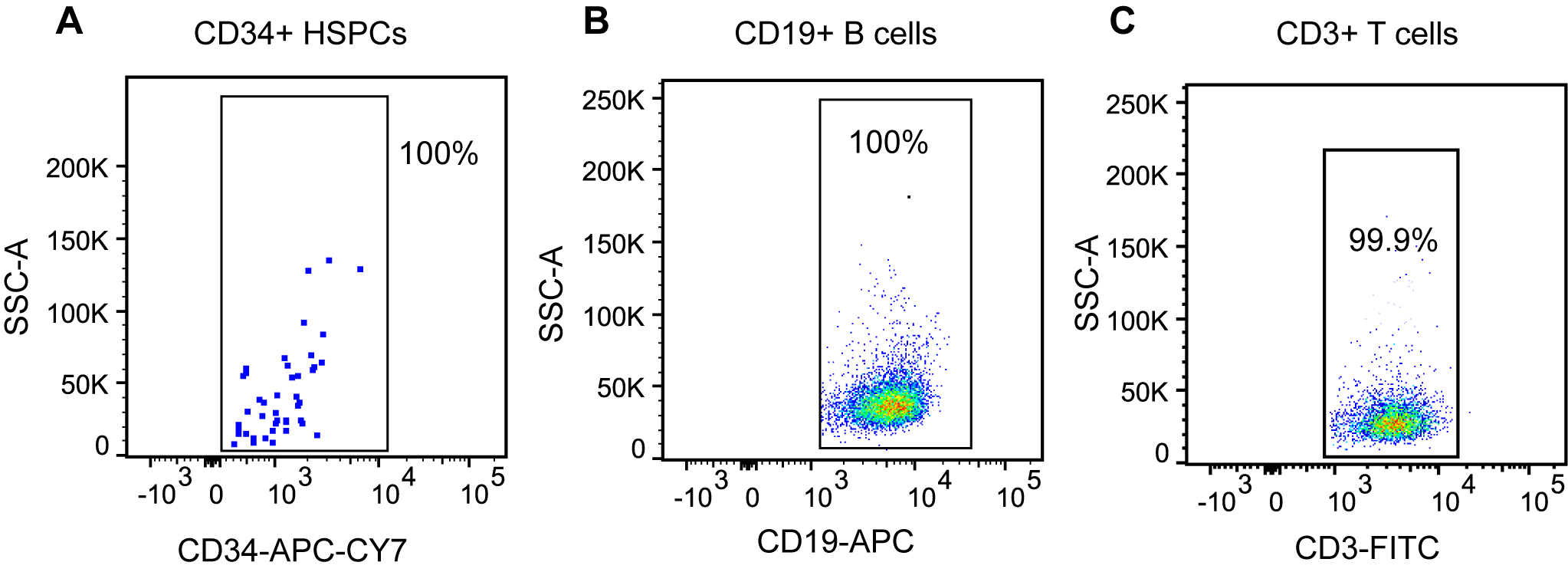


**Supplementary Figure 2**. Dot plot for the determination of three hematopoietic cells purification by flow cytometry. (A) CD34-APC-CY7 analysis of HSPCs; (B) CD19-APC analysis of T cells; (C) CD3-FITC analysis of T cells.

**
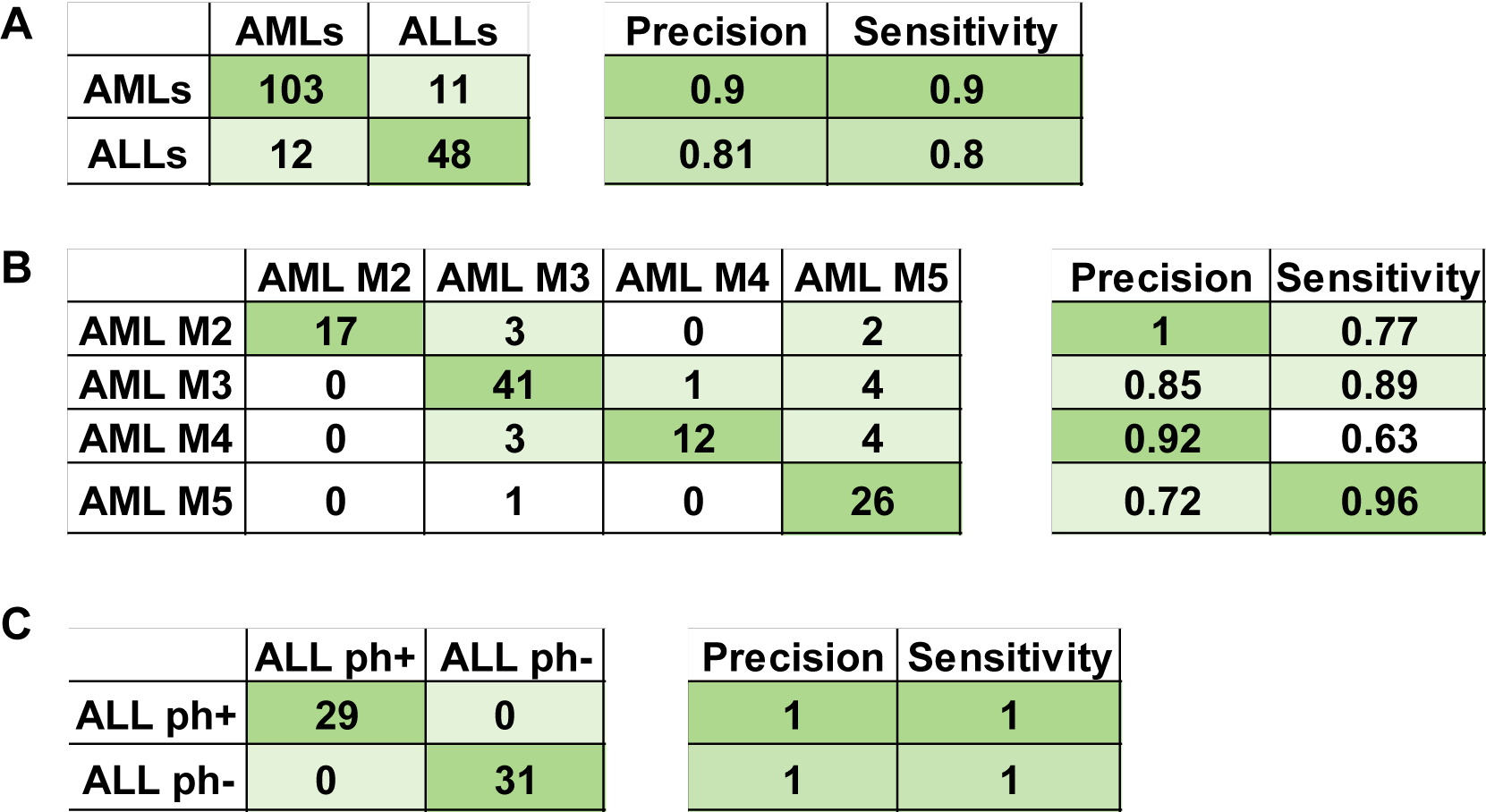
**

**Supplementary Figure 3.** The precision and sensitivity of classification results based on the cell pixel distributions across clusters. (A) “AMLs+ALLs”, (B)“AMLs”, (C)“ALLs”.


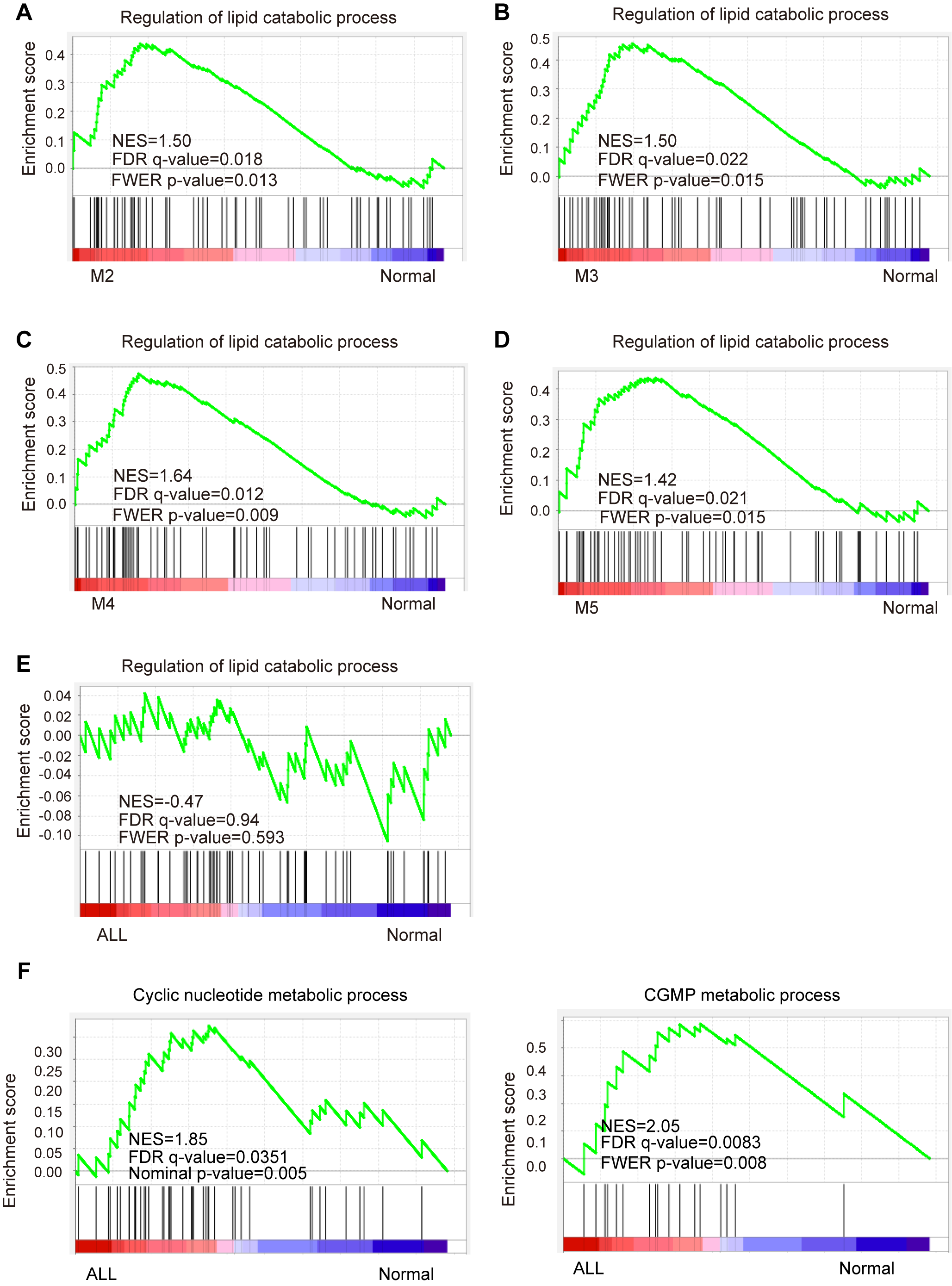


**Supplementary Figure 4**. GSEA of metabolic process of AML and ALL subtypes. The lipid catabolic process across AML subtypes and normal groups (A-E). (A) M2 vs. normal, (B) M3 vs. normal, (C) M4 vs. normal, (D) M5 vs. normal, (E) ALL vs. normal, Normalized enrichment scores (NES), false discovery rate (FDR) q-values, and family-wise error rate (FWER) p-values are indicated in each panel. FDR q-values<0.05 indicate significantly enrichment of lipid catabolic regulation pathways in AML cells compared with controls, while FDR q-values>0.05 (e.g., in E) suggest no significantly enrichment of lipid catabolic regulation pathways. (F) GSEA plots showing enrichment of cyclic nucleotide metabolic process and cGMP metabolic process in ALL compared to normal B cells.


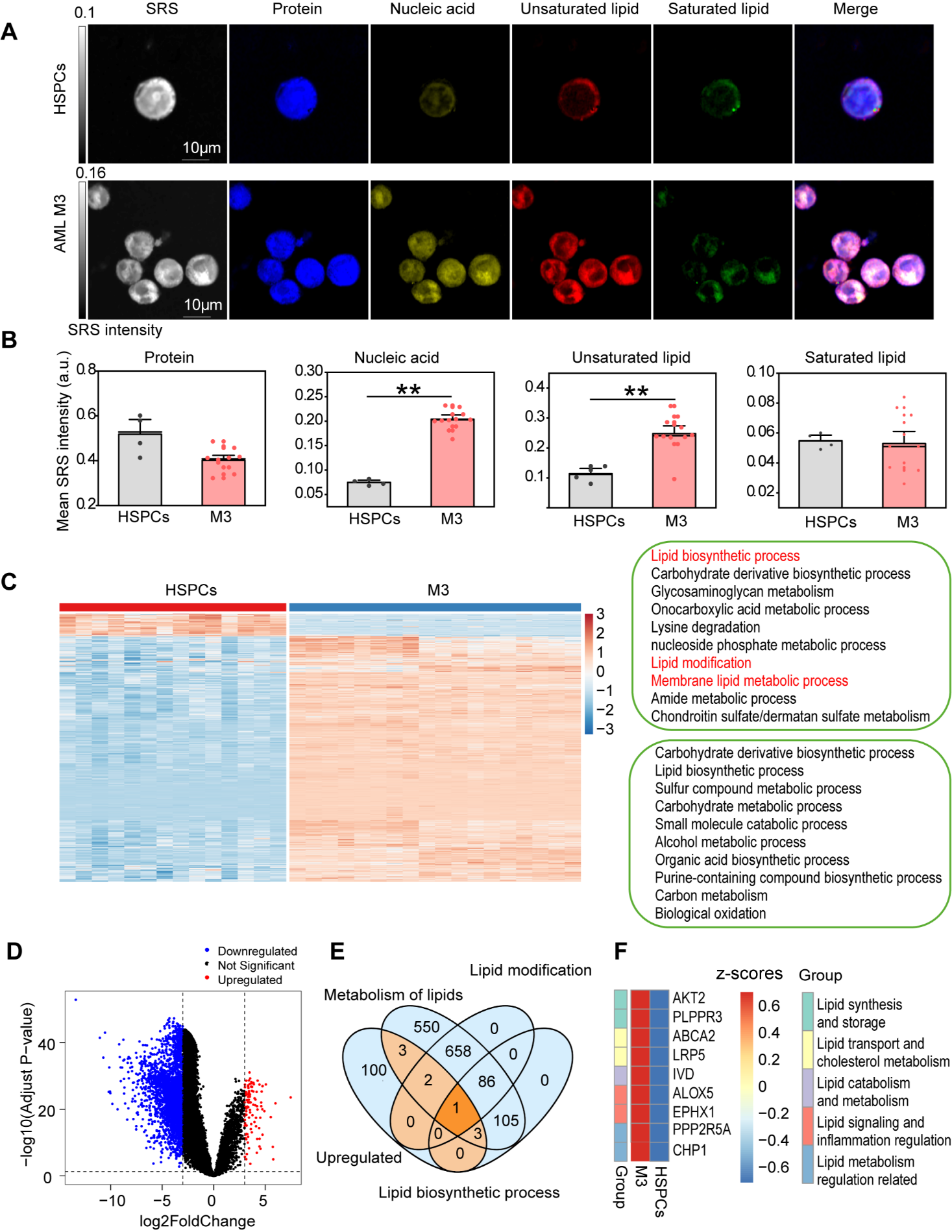


**Supplementary Figure 5**. Comparative H-SRPI imaging and transcriptomic analysis in M3-AML cells versus HSPCs. (A) Representative H-SRPI and multichannel images of HSPCs and M3-AML cells. SRS imaging reveals distributions of proteins (blue), nucleic acids (yellow), unsaturated lipids (red), and saturated lipids (green). (B) Quantification of mean intensity for each biomolecular component (protein, nucleic acid, unsaturated lipid, saturated lipid). P < 0.01, by unpaired two-tailed *t*-test. (C) Heatmap of differentially expressed genes between HSPCs and M3-AML samples. Metascape enrichment analysis of upregulated genes shows prominent involvement of lipid-related pathways. (D) Volcano plot of transcriptomic data comparing HSPCs and M3-AML cells. Red dots indicate significantly upregulated genes, blue dots downregulated genes (FDR < 0.05). (E) Venn diagram showing the intersection of 109 upregulated genes with lipid-related pathways: metabolism of lipids, lipid biosynthetic process, and lipid modification. Nine genes were shared. (F) Heatmap of selected lipid metabolism–related genes enriched in M3-AML cells, grouped by functional category: lipid synthesis and storage, lipid transport and cholesterol metabolism, lipid signaling and inflammation, and lipid metabolism regulation.


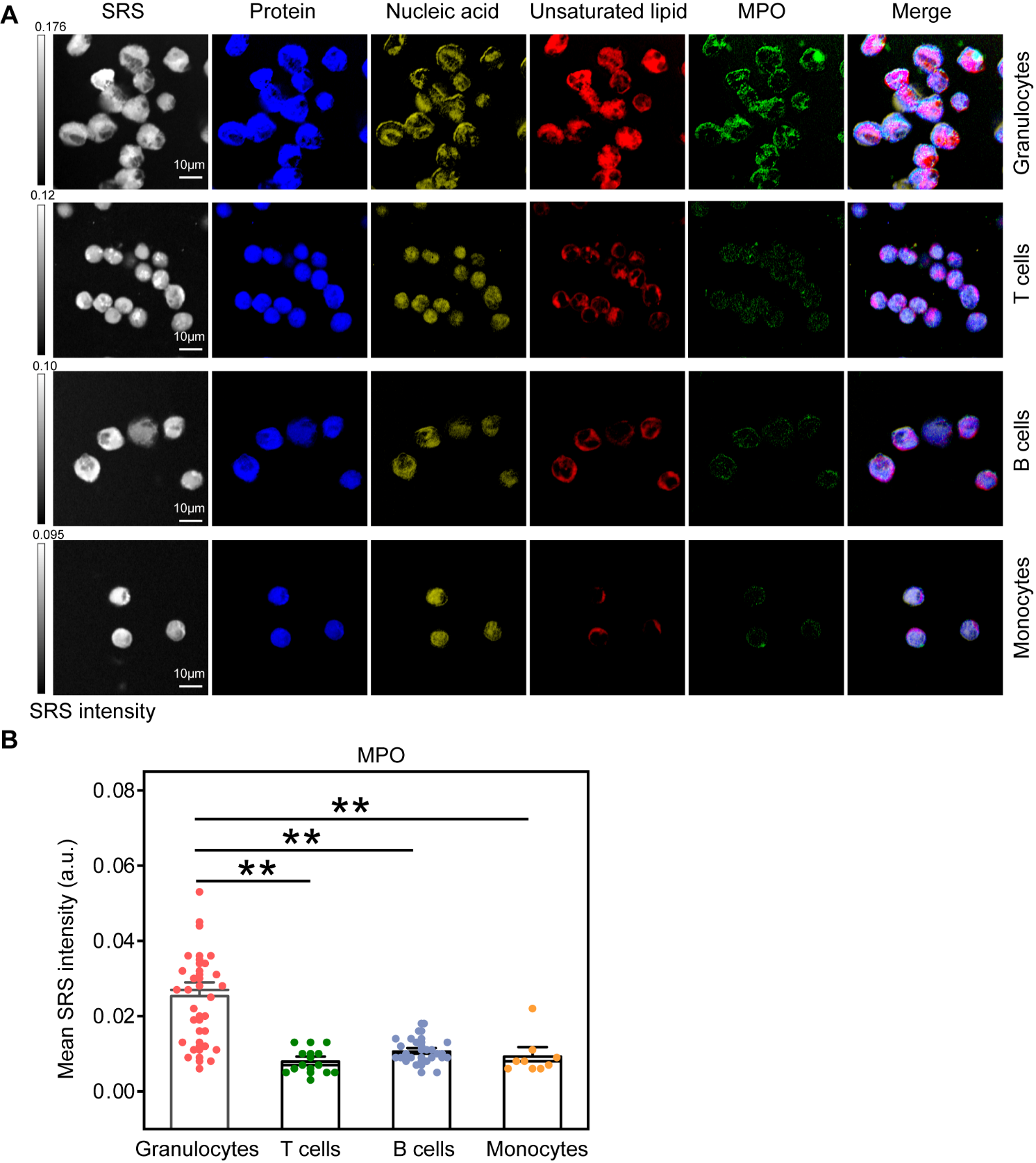


**Supplementary Figure 6.** H-SRPI imaging and quantitative analysis of MPO expression in human immune cell populations. (A) H-SRPI images of granulocytes, T cells, B cells, and monocytes. From left to right,columns display total SRS signal, proteins (blue), nucleic acids (yellow), unsaturated lipids (red), myeloperoxidase (MPO, green), and merged images. (B) Quantification of MPO expression across cell types. Data are presented as mean ± SEM; **p < 0.01, *p < 0.001 by one-way ANOVA with Tukey's post hoc test.

## Supplementary Tables

**Supplemental Table 1.** Characteristics of selected leukemia patients.

| **Gender** | **Age** | **Genetic feature** | **Morphologic Subtype** |
| --- | --- | --- | --- |
| M | 39 | CEBPA,BZIP | M2-AML |
| M | 45 | CEBPA,GATA2,IKZF1,KMT2C | M2-AML |
| F | 67 | PML::RARa(L)+,t(15;17) | M3-AML |
| F | 35 | PML::RARa(L)+,t(15;17) | M3-AML |
| F | 32 | NPM1 mutation | M4-AML |
| M | 23 | FLT3-ITD,CEBPA,BZIP | M4-AML |
| F | 38 | FLT3-ITD,t(9;11) | M5-AML |
| F | 54 | MLL::AF9(+) | M5-AML |
| M | 37 | BCR::ABLP190+,t(9;22) | B-ALL (Ph+) |
| M | 29 | BCR::ABLP190+,t(9;22) | B-ALL (Ph+) |
| M | 25 | EP300::ZNF384 | B-ALL (Ph-) |
| F | 30 | MLL::AF4(+), | B-ALL (Ph-) |

FAB subtypes names, M1: AML without maturation; M3: acute promyelocytic leukemia; M5: acute monoblastic and monocytic leukemia; B-ALL:B-acute lymphoblastic leukemia.

**Refference**

1. Du J, Su Y, Qian C, Yuan D, Miao K, Lee D, et al. Raman-Guided Subcellular Pharmaco-Metabolomics for Metastatic Melanoma Cells. *Nat Commun* (2020) 11(1):4830. Epub 2020/09/26. doi: 10.1038/s41467-020-18376-x.
